# A case of sequential medical therapy for advanced ureteral cancer in Li–Fraumeni syndrome

**DOI:** 10.1002/iju5.12607

**Published:** 2023-07-13

**Authors:** Minami Une, Ryo Fujiwara, Arisa Ueki, Ryosuke Oki, Tetsuya Urasaki, Kentaro Inamura, Shunji Takahashi, Junji Yonese, Takeshi Yuasa

**Affiliations:** ^1^ Department of Genitourinary Oncology, Cancer Institute Hospital Japanese Foundation for Cancer Research Tokyo Japan; ^2^ Department of Clinical Genetic Oncology, Cancer Institute Hospital Japanese Foundation for Cancer Research Tokyo Japan; ^3^ Department of Medical Oncology, Cancer Institute Hospital Japanese Foundation for Cancer Research Tokyo Japan; ^4^ Department of Pathology, Cancer Institute Hospital Japanese Foundation for Cancer Research Tokyo Japan

**Keywords:** advanced urothelial cancer, avelumab, immune checkpoint inhibitor, Li–Fraumeni syndrome, *TP53* pathogenic variant

## Abstract

**Introduction:**

Li–Fraumeni syndrome, an autosomal dominant cancer predisposition syndrome caused by a pathogenic variant of *TP53*, a tumor suppressor gene, leads to a high risk from early childhood of developing various types of cancers. Here, we report a case of advanced ureteral cancer in Li–Fraumeni syndrome.

**Case presentation:**

A 73 years‐old female patient, who had been diagnosed genetically as Li–Fraumeni syndrome; suffered from chondrosarcoma in the left pelvic joint, bilateral breast cancer, endometrial cancer, gastric cancer, and colon cancer in her history. She was diagnosed as unresectable advanced urothelial cancer during continuous magnetic resonance imaging surveillance, underwent avelumab maintenance therapy after the combination of gemcitabine and cisplatin chemotherapy. The efficacies of gemcitabine and cisplatin chemotherapy and avelumab maintenance therapy were good.

**Conclusion:**

We report an advanced urothelial cancer in a patient with Li–Fraumeni syndrome who demonstrated good efficacies to sequential medical therapy.

**Abbreviations & Acronyms**
CT: computed tomography
GC: gemcitabine plus cisplatin
LFS: Li–Fraumeni syndrome
MRI: magnetic resonance imaging
PD‐L1: programmed death‐ligand 1
UC: urothelial cancer

**Keynote message**

We report a case of advanced urothelial cancer in a patient with Li–Fraumeni syndrome treated with sequential gemcitabine plus cisplatin chemotherapy and avelumab maintenance therapy, who demonstrated a radiological partial response. The information on current medical therapy, which includes immune checkpoint inhibitor therapy, for advanced urothelial cancer in Li–Fraumeni syndrome is valuable.

## Introduction

LFS, a cancer predisposition syndrome caused by a pathogenic variant of *TP53*, in known with a high risk from early childhood of developing various types of malignancies.^1^, ^2^, ^3^ A tumor suppressor gene, *TP53*, is considered to be one of the genes responsible for invasive UC.^4^ However, information and frequency of UC in LFS is poor. The Japanese Society for Hereditary Tumors reported that only one (1.5%) patient had bladder cancer.^5^ However, the age at which patients are highly susceptible for UC seems to occur infrequently for UC in LFS. Here, we report a case of advanced ureteral cancer in LFS.

## Case presentation

A 73‐year‐old female patient who had been diagnosed with LFS previously was referred to urology department. She had a left pelvic mass (4.8 × 4.6 × 5.7 cm) and left hydronephrosis was observed using MRI during the surveillance protocol. She underwent a CT‐guided biopsy from left pelvic mass. The pathological diagnostic procedure revealed UC with positive staining for uroplakin and PD‐L1 (5%).

**Fig. 1:**
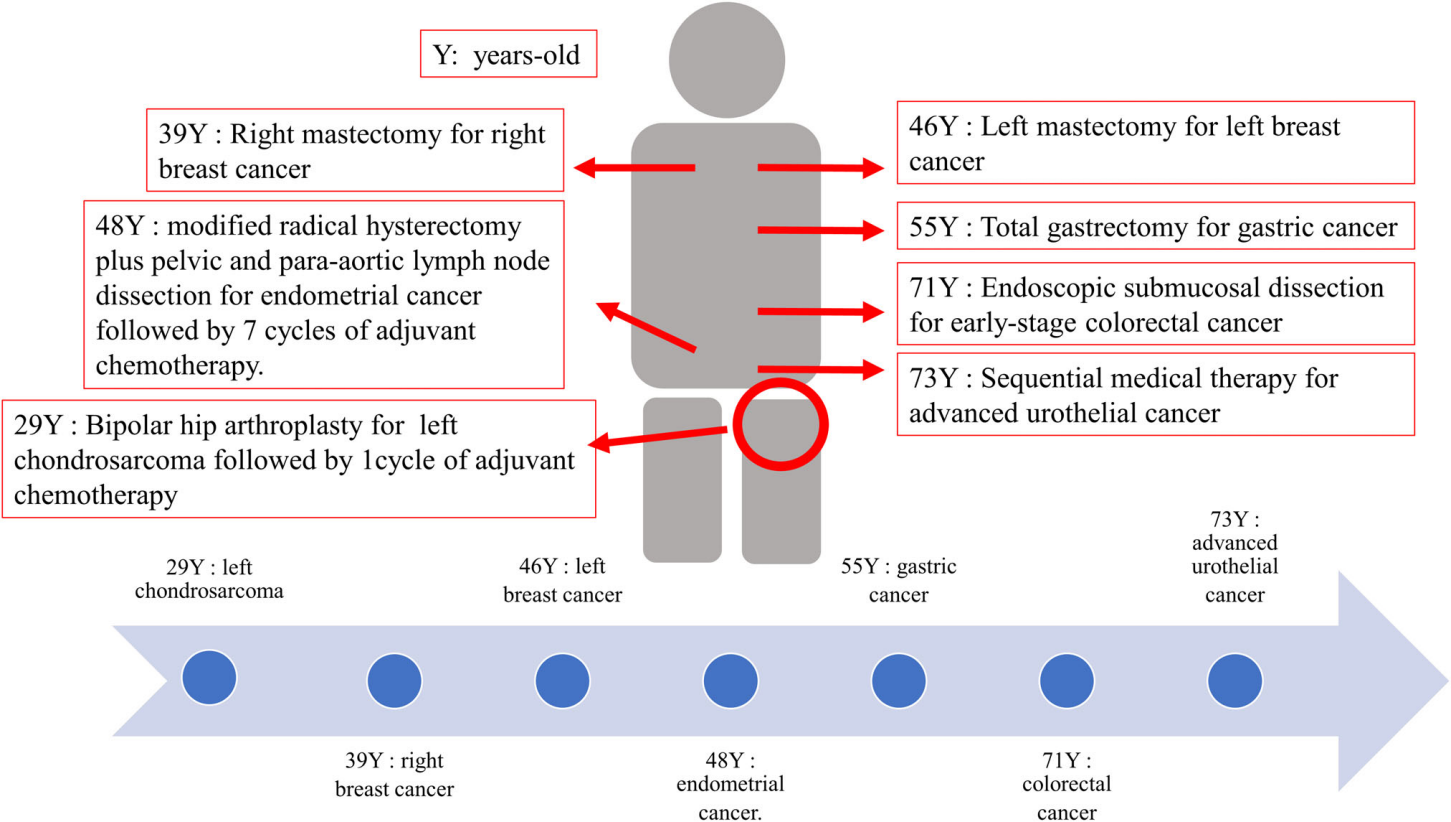
Schematic presentation of the clinical history for various malignancies in this case.

UC was the sixth malignant neoplasm diagnosed during her lifetime. Her first diagnosis of malignant disease was left chondrosarcoma at the age of 29, and she underwent bipolar hip arthroplasty followed by one cycle of adjuvant chemotherapy (doxorubicin plus cyclophosphamide). After that, right and left breast cancer was discovered at 39 and 46 years old, respectively. At the age 48, she underwent a modified radical hysterectomy with pelvic and para‐aortic lymph node dissection for endometrial cancer, followed by seven cycles of an adjuvant chemotherapy (ifosfamide, epirubicin, plus cisplatin). At the age of 55, she underwent a total gastrectomy for gastric cancer. Two primary multiple gastric adenocarcinomas were discovered (Fig. 1).

Due to the repeated multiple malignancies at a relatively young age, genetic counseling was recommended following gastrectomy. As a result, genetic analysis revealed a germline pathogenic variant of *TP53*, and she was genetically diagnosed with LFS. Surveillance was then initiated according to Tront protocol as follows^6^: colonoscopy every 2 years, gastroscopy once a year, whole‐body divided into 3 parts MRI once a year, and abdominal ultrasonography once every 6 months. To avoid radiation‐induced secondary carcinogenesis, imaging retrieval was evaluated by MRI rather than CT.

**Fig. 2:**
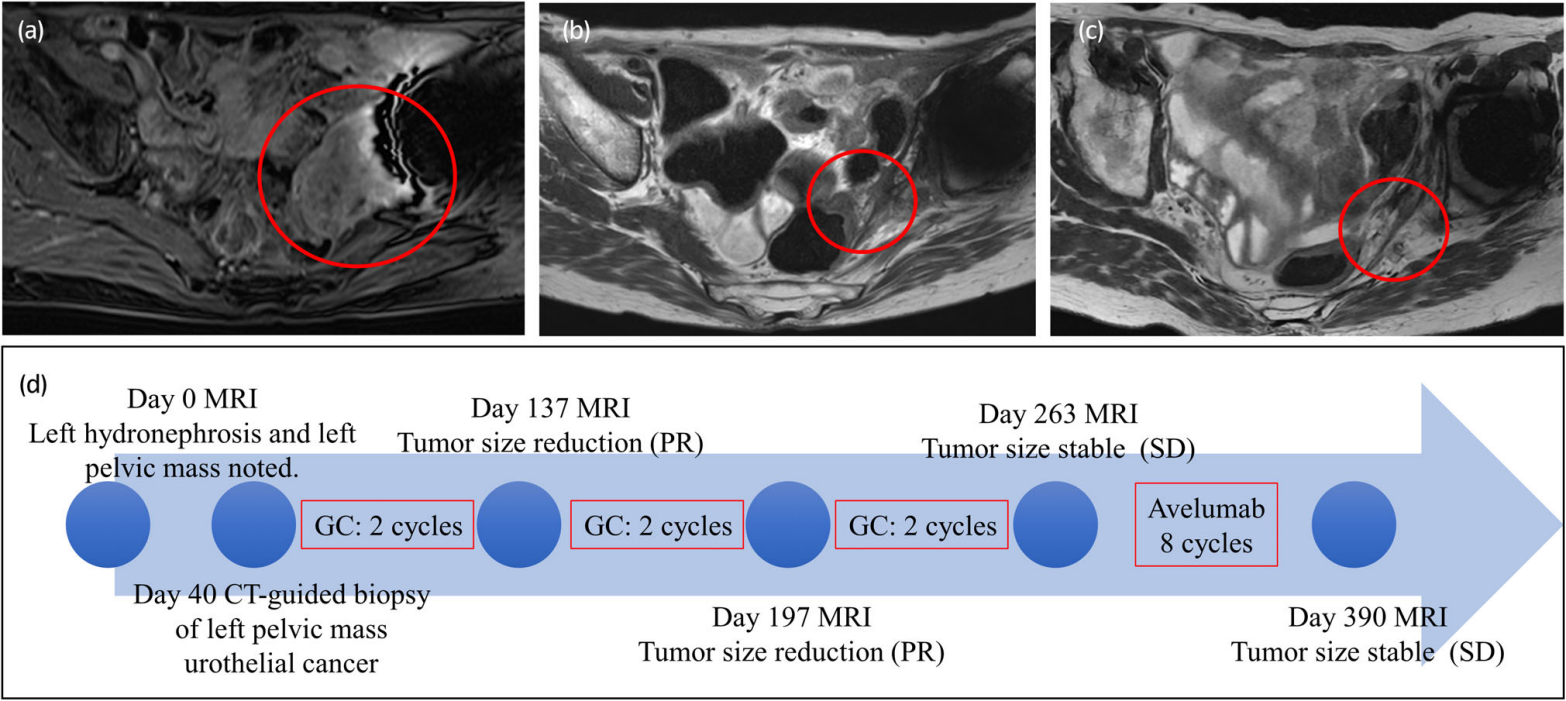
An advanced ureteral cancer in LFS. MRI presentation of advanced ureteral cancer at initial diagnosis (a), after six cycles of the combination of gemcitabine and cisplatin (b), and after eight cycles of avelumab maintenance therapy (c). Schematic presentation for diagnosis and medical therapy for advanced ureteral cancer (d).

During the surveillance protocol, transverse colon cancer was detected by colonoscopy and resected by endoscopic submucosal dissection. Two years after the occurrence of colon cancer, left UC, which invaded the pelvic wall, was diagnosed by MRI (Figs 1 and 2a). Because surgical removal was considered to be impossible, she underwent systemic chemotherapy, which consisted of GC. At the end of the second course, the tumor was evaluated by MRI and determined to be a partial response (PR: 80% regression). As the size of her tumor shrank during the six cycles of GC therapy, she initiated avelumab maintenance therapy (Fig. 2b). Four months after the initiation of avelumab maintenance therapy, her left pelvic tumor remained shrunken, and no new metastatic lesions have appeared to date (Fig. 2c,d).

## Discussion

Characteristics of Japanese LFS have been reported previously.^5^ Sixty eight patients possessed the *TP53* germline variant (34 females and 34 males) and a total of 128 tumors developed in these patients. The median age at first tumor onset was 16.5 years (range: 8 months to 65 years). Multiple primary cancers (*n* = 2–7) were observed in 32 of the 68 affected patients (47.0%). The most frequent primary malignancy was breast cancer (36.8%) followed by bone tumors (23.5%), brain tumors (17.6%), hematologic malignancy (16.2%), soft tissue sarcoma (14.7%), gastric cancer (14.7%), lung cancer (14.7%), colorectal cancer (14.7%), adrenocortical cancer (13.2%), hepatocellular cancer (5.9%), and other cancers (14.7%).^5^ In the other malignancies, one kidney cancer (1.5%) and one bladder cancer (1.5%) were diagnosed.^5^ Only two (2.9%) urological malignancies were reported besides adrenocortical cancer. The median age of adrenocortical cancer onset was 3 years old (range: 0–31 years old).^5^ Therefore, despite the strong association between *TP53* and invasive UC, Japanese urologists seem to be unfamiliar with LFS.

The changes in *TP53* represent one of the most common genetic events in patients with invasive UC. Paradoxically, the alterations in *TP53* have been linked not only with carcinogenesis and progression, but also to chemosensitivity.^4^ It was demonstrated that patients who had a mutated somatic *TP53* gene responded significantly better to the cisplatin‐based systemic chemotherapy than those with the wild‐type *TP53* gene in a neo‐adjuvant setting.^7^

Currently, after the failure of first‐line GC therapy, pembrolizumab is the standard second‐line therapy.^8^, ^9^ Conversely, when GC therapy demonstrates good efficacy, avelumab is used as maintenance therapy.^8^, ^9^

*TP53* plays a role not only as a tumor suppressor but also in immune system regulation. As TP53 is one of the key factors in the DNA damage repair pathway, TP53 mutation was associated with increased tumor mutational burden (TMB). In addition, clinical benefit with immunotherapy is considered to be greatest among those with the highest mutation burden.^10^, ^11^, ^12^ Therefore, the implications of the *TP53* mutation when predicting the response to immune checkpoint inhibitor therapy.^13^, ^14^ Olivares‐Hernández reported that patients with lung adenocarcinoma with *TP53* mutation had a greater response to immune checkpoint inhibitors in progression‐free survival and overall survival.^14^ In this case, our patient demonstrated a PR to first‐line GC therapy, followed by avelumab maintenance therapy for more than 4 months.

We report a case of advanced UC in LFS, for a patient treated with GC therapy followed by avelumab maintenance therapy. To the best of our knowledge, this case is the first example of modern medical therapy for UC in LFS.

## Author contributions

Minami Une: Investigation; methodology; writing – original draft. Ryo Fujiwara: Investigation; methodology; project administration; writing – original draft; writing – review and editing. Arisa Ueki: Methodology; validation; writing – review and editing. Ryosuke Oki: Methodology; writing – review and editing. Tetsuya Urasaki: Methodology; writing – review and editing. Kentaro Inamura: Methodology; writing – review and editing. Shunji Takahashi: Methodology; writing – review and editing. Junji Yonese: Methodology; writing – review and editing. Takeshi Yuasa: Investigation; methodology; project administration; supervision; validation; writing – original draft; writing – review and editing.

## Conflict of interest

The authors declare no conflict of interest.

## Approval of the research protocol by an Institutional Reviewer Board

Not applicable.

## Informed consent

An informed consent from the patient was obtained.

## Registry and the Registration No. of the study/trial

Not applicable.
